# Patient satisfaction with chronic disease care and its associated factors in primary health care facilities in Johannesburg, South Africa

**DOI:** 10.3389/frhs.2023.967199

**Published:** 2023-05-16

**Authors:** Juliana Kagura, Natasha Khamisa, Zvifadzo Matsena Zingoni, Neo Dulaze, Yaw Awuku-Larbi, Ndumiso Tshuma

**Affiliations:** ^1^ Division of Epidemiology and Biostatistics, School of Public Health, University of the Witwatersrand, Johannesburg, South Africa; ^2^ Division of Health and Society, School of Public Health, Faculty of Health Sciences, University of Witwatersrand, Johannesburg, South Africa; ^3^ Division of Bioethics and Health Law, School of Clinical Medicine, Faculty of Health Sciences, University of Witwatersrand, Johannesburg, South Africa; ^4^ The Best Health Solutions, Johannesburg, South Africa

**Keywords:** patient satisfaction, chronic care, factor analysis, level of satisfaction, Johannesburg

## Abstract

**Background:**

Patient satisfaction is a widely used indicator of assessing health care quality and has been used by policymakers to consider the needs of patients when developing suitable strategies for safe and high-quality care. However, in South Africa, the dual burden of HIV and NCDs has implications for the health system, whereby the factors influencing the quality of care and patient satisfaction may be unique to this context. Thus, this study examined the predictors affecting chronic disease patients' levels of satisfaction with care in Johannesburg, South Africa.

**Methods:**

A cross-sectional study was conducted among 2,429 chronic disease patients at 80 primary healthcare facilities in Johannesburg, South Africa. A questionnaire derived from existing literature and patient satisfaction frameworks was used to measure the level of satisfaction of patients when receiving care. Patients' overall satisfaction was categorized into not satisfied and satisfied. Cronbach's alpha was used to assess scale reliability. Factor analysis was used as a data dimension reduction approach and the Kaiser-Meyer-Olkin and the Bartlett test of sphericity were used to test the sampling adequacy and to examine the inter-independence of the items. Logistic regression was used to determine factors associated with being satisfied. Significance was set at 5%.

**Results:**

The majority of chronic disease patients 65.5% (*n* = 1,592) were aged 18−30 years; 63.8% (*n* = 1,549) were females, 55.1% (*n* = 1,339) were married and 2,032 (83.7%) were satisfied with care. The factor analysis results were in five sub-scales namely improving values and attitudes, cleanliness of the clinic, safe and effective care, infection control, and on the availability of medicines. In adjusted models, patients aged >51years had an increased odds of 3.18 (95% CI:1.31−7.75) of being satisfied compared to those aged 18−30 years and patients who had visited the clinic at least 6 times had 51% increased odds of being satisfied (AOR = 1.51,95% CI:1.13–2.03). The odds of being satisfied increased by 28% (AOR = 1.28,95% CI:1.07–1.53), 45% (AOR = 1.45,95% CI:1.2–1.75), 34% (AOR = 1.34,95% CI:1.13–1.59) and 4.31 (95% CI:3.55–5.23) for every score increase in the factors like improving values and attitudes, cleanliness of clinic safe and effective care and availability of medicine, respectively.

**Conclusions:**

Key predictors of patient satisfaction were found to be sociodemographic factors including age, distance to the clinic, number of visits and waiting times as well as factors such as improving values and attitudes, cleanliness of the clinic, waiting time, safety and effective care and availability of medicines. Adjustment of existing frameworks for addressing context-specific improvement of patient experiences such as security and safety is recommended to ensure healthcare quality and service utilization for better chronic disease outcomes in South Africa.

## Introduction

Chronic diseases are defined as diseases that require continuous treatment management for a lifetime. These include infectious diseases like HIV/AIDS and tuberculosis (TB) (1); and non-communicable diseases (NCDs) like hypertension, and type II diabetes mellitus (2). The management of these requires an efficient and enforced healthcare system for ensuring the utilization of healthcare services and subsequently, patient satisfaction (3). Based on global population averages, chronic diseases were projected to increase to 73% by 2020 (4).

There is a particularly high burden of HIV/AIDS and NCDs in South Africa and this has serious implications for its health system (1). Diabetes Mellitus has been reported as the second leading underlying cause of death, with its proportion increasing from 5,4% in 2015 to 5,7% in 2017 (5). As of 2019, 4.74 million South Africans were suffering from hypertension, making it the most prevalent chronic health condition in the country (6). The implications of an increase in chronic disease patients mean an increase in healthcare visits and treatment costs (estimated at 25 billion between 2006 and 2015) (7). This justifies the importance of providing quality health services to improve uptake as well as patient satisfaction, towards reducing chronic disease mortality rates.

Patient satisfaction is a widely used indicator of assessing health care quality (8) and has been used by policymakers to consider the needs of patients when developing suitable strategies for safe and higher-quality care. However, in South Africa, the dual burden of HIV and NCDs has implications for the health system, whereby the factors influencing the quality of care and patient satisfaction may be unique to this context (1). A South African study conducted in Free State reported high overall satisfaction with general and nurse-provided services among patients receiving public-sector ART but the waiting time reduced patient satisfaction (9). Another study reported that both race and SES were significant predictors of levels of satisfaction with the services of the healthcare provider. The white race and high SES respondents were more likely to report excellent service (10). Furthermore, patient safety in the context of South Africa bears a different meaning with 60% of health personnel experiencing workplace crime and violence. This was confirmed in another similar study which showed that 76% of health workers felt that the security systems in their workplace were poor and that this made them feel unsafe (11). This is not well understood in relation to patient satisfaction with a scarcity of studies available among patients within this context.

In response to the high burden of chronic diseases in South Africa, there have been context-specific interventions such as the integrated chronic disease management (ICDM) model introduced in 2011 to improve the quality of care and health outcomes of patients (1). The key priorities include improving the values and attitudes of staff, cleanliness of facilities, patient safety and security, infection prevention and control as well as the availability of medicines which are factors associated with patient satisfaction (12).

As an important aspect of healthcare quality, patient satisfaction indicates that the healthcare provider is successful in meeting the patients' needs (13). Patient satisfaction is believed to provide insight into safety, accessibility, equity and comprehensiveness of quality care (14) but patient experience is often overlooked as a part of this. For example, patient satisfaction includes preferences such as male/female healthcare providers or rights such as the right to respect (3). The 5P model is one of the few frameworks providing a holistic view of the patient experience using five dimensions namely provider, physician, patient, personnel and periphery which contribute to patient satisfaction (15). This renders patient satisfaction a complex and multifaceted concept with several determining factors like health care-provider related factors (technical care, interpersonal care), physical environment, access (accessibility, availability, and affordability), organisational characteristics (continuity of care, and outcome of care); and patient-related factors (age, gender, education, socio-economic status, marital status, race, religion, geographic characteristics, visit regularity, length of stay, health status, personality, and expectations) (16–18). Measuring patient satisfaction, therefore, requires a more holistic approach. The WHO Quality of Care Framework based on optimal health, responsiveness and fairness in financing as well as the Bamako Initiative which is more economically oriented to achieve a quality of care, both agree on the complex and multi-dimensional nature of healthcare quality and patient satisfaction makes it difficult to measure using a standardised approach (19). In line with the Donabedian model, this complexity is exacerbated by the varying nature of healthcare as well as the various stakeholders and their vested interests, thus requiring the adaptation of existing frameworks in understanding different health systems and needs (20).

Other popular existing frameworks for measuring health care quality and patient satisfaction are centred upon aspects of service quality including tangibles such as physical environment as well as empathy and individualized care from providers (21). The Service Quality Management Scale (SERVQUAL) is developed using this understanding and has been successfully used in assessing patient satisfaction in hospital settings (21). However, the significant diversity in the conceptualization of patient satisfaction determinants compromises the utilization of the SERVQUAL tool across contexts. Studies have shown that the location where patient satisfaction surveys are conducted as well as the methodological approach used to assess satisfaction is very likely to influence respondents’ expression of lived experiences with health care (1).

Johannesburg, South Africa has been reported as one of the metropolitan areas currently experiencing major challenges with 78% of patients being dissatisfied with the quality of chronic healthcare received (22). Further implications include negative effects on health outcomes, with dissatisfied chronic disease patients being less likely to utilize health services or adhere to medicine compared to satisfied chronic patients who were more likely to comply (23–25).

This study seeks to determine the context-specific factors influencing patient satisfaction among hypertension and diabetes chronic disease patients in Johannesburg, South Africa. Understanding patients' experiences in relation to patient satisfaction is necessary for providing clear direction for the development of appropriate strategies aimed at improving the quality of services delivered (26).

## Methods

### Study design and site

Secondary data analysis of a quantitative, analytical, cross-sectional design was conducted in the City of Johannesburg from January to December 2016. The City of Johannesburg is composed of 7 regions covering an area of 1645 square kilometres with a population of approximately 4 million people. The City of Johannesburg has a total of 80 primary health care (PHC) facilities which are the lowest level of patient care in the hierarchy of tiers of health care. These provide interventions and services like preventive services for maternal, newly-born, children, adults, and geriatrics; diagnostic services of radiology and pathology services; treatment of essential drugs of consultative services, the medical and surgical procedure of devices and consumables; rehabilitation and palliative services (27).

### Study population and sample size

This study targeted all patients who were managed for chronic disease in the PHC facilities in the City of Johannesburg aged 18 years and above. A sample size of 2,429 participants was considered in this study from 80 PHCs which implemented chronic care disease clubs. Since this was a secondary data analysis for programme data, no sample size was calculated but all patients who met the inclusion criteria were included in the study. Patients who were less than 18 years and those who were in emergency cases were excluded from this study.

### Data collection and tool validity

The primary study used an interview-administered questionnaire with structured questions to collect data from all study participants electronically. The questionnaire consisted of a total of 22 items derived from existing literature (11) around important aspects of patient satisfaction such as the availability of medicines and cleanliness of facilities. The items were also developed using holistic frameworks focusing on patient experiences related to safe and effective care as well as attitudes and values (28). Furthermore, specific items related to safety and security were included within the questionnaire to measure context-specific factors related to patient satisfaction (12). In addition, sociodemographic characteristics derived from patient-related factors associated with patient satisfaction in the literature (11) were measured on a five-point Likert-type scale defined as 1 = strongly disagree, 2 = disagree, 3 = neutral/I don't know, 4 = agree, and 5 = strongly agree. The test of reliability for each item was performed using Cronbach's alpha test and a score of 0.7 was considered the reliable threshold.

### Outcome variable

The patients' overall satisfaction was measured on a Likert scale defined as 1 = strongly disagree, 2 = disagree, 3 = neutral/I don't know, 4 = agree, and 5 = strongly agree. A binary outcome was generated by categorising strongly disagree, disagree and neutral/I don't know as not satisfied (coded 0) and combining agree and strongly agree into satisfied (coded 1).

### Independent variables

In this study, socio-demographic characteristics (age groups (18–30, 31–40, 41–50, > 50 years), sex (male, female), marital status (never married, married), employment status (not employed, employed), distance to the clinic (within 5Km, > 5Km), service points (ART, chronic), number of clinic visits (< 6 times, ≥ 6 times) and waiting period (<1 h, 1–2 h, 3–4 h, 5–6 h, > 6 h) were the covariates collected. The sub-scales (factors) from the factor analysis dimension reduction were also considered independent variables in the regression analysis. These include improving values and attitudes (factor of priority 1) defined by 4 items (such as “I was treated with respect and dignity by the staff at this clinic”), cleanliness of clinic (factor of priority 2) defined by 5 items (such as “the toilets are clean at this clinic”), safe and effective care (factor of priority 3) defined by 5 items (such as “there is a security guard who is visible at all times at this clinic”), infection control (factor of priority 4) defined by 4 items (such as “there are no visible soiled gloves, swabs, syringes or needles lying around in this clinic”) and availability of medicines (factor of priority 5) defined by 3 items (such as “all the prescribed medicines were available at this clinic”).

### Data analysis

Collected data were imported into STATA version 16. All data management and analysis were also performed in Stata. Descriptive statistics were reported using frequencies and percentages for categorical data and median and interquartile range (IQR) for continuous non-normal data. Bivariate analyses were performed for all baseline characteristics stratified by patient satisfaction. Additionally, proportions were compared using the Chi-square test. Factor analysis was used to assess the dimensions of the patient's satisfaction with the quality of service provided. The statistical criteria Kaiser-Meyer-Olkin (KMO)-whereby a value close to 1 is considered favourable- and the Bartlett test of sphericity- a *p*-value *p* < 0.05 is favourable-were used to test the sampling adequacy and to examine the inter-independence of the subscales of the scale. To compute factor loadings, orthogonal rotation was used. The average variance extracted (AVE), average factor loading (AFL) values, the correlation matrix square (CMS) and the average variance extracted (AVE) for each sub-scale (factor) were also estimated. Construct validity was established using AFL.

Univariate and multiple logistic regressions were performed to identify factors associated with patients' satisfaction and quantify the effect of each factor on the patient's satisfaction. All the variables used in the univariate analysis were considered in the adjusted model. The model goodness of fit was assessed using the Hosmer and Lemeshow test. The odds ratios (OR) and adjusted odds ratio (AOR) measures of association and their corresponding 95% confidence intervals (CI) were reported for each covariate. The significance of each coefficient was tested by the Wald test, and statistical significance was considered at *p* < 0.05.

### Ethics

Ethical clearance for this study was obtained from Monash University, South Africa (approval number: 9693).

## Results

### Socio-demographic factors

A total of 2,429 chronic disease patients participated in this study. Table 1 below summarises the socio-demographic factors of these participants. The overall number of chronic disease patients who were not satisfied with care was 397 (16.3%). The majority of the chronic disease patients 65.5% (*n* = 1,592) fell in the 18–30 years category; 63.8% (*n* = 1,549) were females, 55.1% (*n* = 1,339) were married and 57.1% (*n* = 1,387) were employed. Most of the patients 62.5% (*n* = 1,518) were staying within a 5 Km distance to the clinic, 55.7% (*n* = 1,353) were receiving chronic disease care; 58.3% (*n* = 1,415) reported having visited the clinic less than 6 times annually and 27.9% (*n* = 677) reported a clinic service waiting time of 3–4 h.

**Table 1:** Baseline characteristics of the patients in the city of Johannesburg.

Characteristics	Overall *n* (%)	Chronic Disease Patients’ Satisfaction with care		*p*-value
		Not satisfied n (%) 397 (16.3)	Satisfied *n* (%) 2,032 (83.7)	
Age group in years				
18–30	1,592 (65.5)	291 (73.3)	1,301 (64.0)	**0**.**001**
31–40	414 (17)	57 (14.4)	357 (17.6)	
41–50	250 (10.3)	36 (9.1)	214 (10.5)	
>51	173 (7.1)	13 (3.3)	160 (7.9)	
Sex				
Female	1,549 (63.8)	261 (65.7)	1,288 (63.4)	0.371
Male	880 (36.2)	136 (34.3)	744 (36.6)	
Marital Status				
Never married	1,090 (44.9)	186 (46.9)	904 (44.5)	0.387
Married	1,339 (55.1)	211 (53.2)	1,128 (55.5)	
Employment Status				
Not Employed	1,042 (42.9)	159 (40)	883 (43.5)	0.21
Employed	1,387 (57.1)	238 (60)	1,149 (56.5)	
Distance to Clinic				
Within 5 km	1,518 (62.5)	229 (57.7)	1,289 (63.4)	**0**.**03**
>5 km	911 (37.5)	168 (42.3)	743 (36.6)	
Service Point				
ART	1,076 (44.3)	171 (43.1)	905 (44.5)	0.591
Chronic	1,353 (55.7)	226 (56.9)	1,127 (55.5)	
Number of Visits in year				
<6 times	1,415 (58.3)	255 (64.2)	1,160 (57.1)	**0**.**008**
≥6 times	1,014 (41.7)	142 (35.8)	872 (42.9)	
Length of waiting time				
<1 h	472 (19.4)	71 (17.9)	401 (19.7)	**<0**.**001**
1–2 hours	851 (35)	110 (27.7)	741 (36.5)	
3–4 hours	677 (27.9)	122 (30.7)	555 (27.3)	
5–6 hours	301 (12.4)	71 (17.9)	230 (11.3)	
>6 hours	128 (5.3)	23 (5.8)	105 (5.2)	

Boldfaced *p*-values are significant at 5%.

There were 2032 (83.7%) who were satisfied with the care they received while 397 (16.3%) of the participants were not satisfied. A bivariate analysis of the patient's satisfaction by socio-demographic characteristics is shown in Table 1 above. Patient satisfaction was significantly associated with age group (*p*-value = 0.001); distance to the clinic (*p*-value = 0.03); the number of visits in the year (*p*-value = 0.0008) and length of waiting time (*p*-value < 0.0001). More patients who were aged 18–30 years (64%), who lived within the 5 Km radius (63.4%), who had visited the clinic at most 6 times a year (57.1%) and reported 1–2 h waiting time (36.5%) were satisfied.

### Exploratory factor analysis

The global KMO for all the patient levels of satisfaction items was 0.901 and the corresponding Bartlett test of sphericity was significant (*p*-value < 0.0001). The KMO and the Bartlett sphericity test showed that there was a substantial correlation in data; hence, the variables were intercorrelated. The Cronbach's alpha for the scale reliability value for the 22 standardized items was 0.917.

Table 2 below summarises the factor loading for each item and the identified sub-scales (factors). The median score per item was 4 and the factor loadings ranged from 0.6–0.8. Convergence reliability was established for 19 items as 3 items had factor loadings below 0.7. All 22 items had a reliability coefficient of at least 0.7.

**Table 2:** The exploratory factor analysis results for the patient's satisfaction items and scale reliability.

Factors	Items	Median (IQR)	Factor loading	Alpha
Factor of priority 1: Improving values and attitudes	I was informed about my rights and responsibilities in a way that was clear and easy to understand at this clinic	4 (3–5)	0.7	0.8
	I was given information about my condition in a way that was clear and easy to understand at this clinic	4 (4–5)	0.8	0.7
	I have been informed of the complaints procedure at this clinic	4 (3–5	0.8	0.8
	I was treated with respect and dignity by the staff at this clinic	4 (4–5)	0.7	0.8
Factor of priority 2: Cleanliness of Clinic	This clinic is clean and presentable	4 (4–5)	0.7	0.8
	The toilets are clean at this clinic	4 (4–5)	0.8	0.8
	There are refuse bins in the waiting area at this clinic	4 (4–5)	0.7	0.8
	The walls are free of dirt and spider webs at this clinic	4 (4–5)	0.7	0.8
	The floor and tables are clear of splashes, spills and stains at this clinic	4 (4–5)	0.7	0.8
	The yard is clean and presentable at this clinic	4 (4–5)	0.6	0.8
Factor of priority 3: Safe and effective care	There is a security guard who is visible at all times at this clinic	4 (4–5)	0.5	0.8
	This clinic is easily accessible to individuals with disabilities	4 (3–5)	0.8	0.8
	There is a visible ramp for wheelchairs at this clinic	4 (3–5)	0.8	0.8
	There is a toilet that is easily accessible to individuals with disabilities at this clinic	4 (3–5)	0.8	0.8
	I feel safe at this clinic	4 (4–5)	0.6	0.8
Factor of priority 4: Infection Control	The toilet floors are free of spills, splashes and stains at this clinic	4 (4–5)	0.8	0.8
	There is adequate running water, toilet paper and soap in the toilets at this clinic	4 (4–5)	0.8	0.9
	This clinic is free of bad odours and smells	4 (4–5)	0.7	0.8
	There are no visible soiled gloves, swabs, syringes or needles lying around in this clinic	4 (4–5)	0.7	0.9
Factor of priority 5: Availability of Medicines	All the prescribed medicines were available at this clinic	4 (4–5)	0.8	0.8
	I was informed on how to take my medications in a way that was clear and understandable at this clinic	4 (4–5)	0.8	0.7
	A nurse clearly explained any medication side effects at this clinic	4 (4–5)	0.8	0.8

Italised are factor loadings < 0.7.

From the factor analysis, 5 sub-scales (factors) were identified which the dimensions are: improving values and attitudes (factor of priority 1) defined by 4 items, cleanliness of clinic (factor of priority 2) defined by 5 items, safe and effective care (factor of priority 3) defined by 5 items, infection control (factor of priority 4) defined by 4 items and availability of medicines (factor of priority 5) defined by 3 items ( Table 2 ).

Table 3 summarises the sub-scale (factors) average factor loadings, correlation, reliability, validity and sampling adequacy. The average Cronbach alpha, KMO, composite reliability and average factor loading were at least 0.7.

**Table 3:** Validity, reliability and sampling adequacy of the patient satisfaction sub-scales.

Factors	Average Cronbach alpha	KMO	Composite Reliability	AFL	AVE	CMS	Bartlette test of sphericity
Factor 1: Improving values and attitudes	0.8	0.8	0.8	0.8	0.5	0.051	<0.0001
Factor 2: Cleanliness of Clinic	0.8	0.8	0.8	0.8	0.5	0.003	<0.0001
Factor 3: Safe and effective care	0.8	0.8	0.8	0.7	0.5	0.012	<0.0001
Factor 4: Infection Control	0.9	0.8	0.8	0.8	0.5	0.014	<0.0001
Facto 5: Availability of Medicines	0.9	0.7	0.8	0.8	0.5	0.062	<0.0001

AVE, average variance extracted; KMO, kaiser-meyer-olkin; AFL, average factor loading; CMS, correlation matrix square.

### Factors associated with chronic disease patients’ satisfaction with care

Factors associated with being satisfied were assessed using logistic regression models and the results are shown in Table 4 below. At the univariate level, patients aged 31−40 years and 51 years and above had an increased odds of 1.4 (95% CI:1.03–1.9) and 2.75 (95% CI:1.54–4.91) of being satisfied compared to those aged 18-30 years, respectively. Patients who lived at least 5 km from the clinic had reduced odds of 21% (OR = 0.79, 95% CI: 0.63–0.98) compared to those who lived within 5 km of the clinic. Patients who reported having visited the clinic at least 6 times in contrast to those who had fewer than 6 visits had 35% increased odds of being satisfied (OR = 1.35, 95% CI: 1.08–1.69). Compared to those who waited for less than an hour, those who waited 1–2 h and 3–4 h had reduced odds of 32% (OR = 0.68, 95% CI: 0.51–0.89) and 52% (OR = 0.48, 95% CI: 0.34–0.67), respectively. The odds of being satisfied increased by 2.32 (95% CI: 2.06–2.6), 2.47 (95% CI: 2.19–2.79), 2.43 (95% CI: 2.16–2.73), 2.21 (95% CI: 1.98–2.46) and 5.55 (95% CI: 4.67–6.6) for every score increase in the improving values and attitude factor, cleanliness of clinic factor, safe and effective car factor, infection control factor and availability of medicine factor, respectively.

**Table 4:** Factors associated with being satisfied with chronic disease patient care.

Characteristics	Univariate models		Adjusted model	
	OR (95% CI)	*p*-value	AOR (95% CI)	*p*-value
Age				
18–30	Ref		Ref	
31–40	1.4 (1.03–1.9)	**0.031**	1.29 (0.88–1.9)	0.186
41–50	1.33 (0.91–1.94)	0.137	1.12 (0.73–1.91)	0.507
>51	2.75 (1.54–4.91)	**0.001**	3.18 (1.31–7.75)	**0.011**
Employment Status				
Not Employed	Ref		Ref	
Employed	0.87 (0.7–1.08)	0.21	0.93 (0.7–1.23)	0.602
Distance to Clinic				
Within 5 km	Ref		Ref	
>5 km	0.79 (0.63–0.98)	**0.031**	1.17 (0.89–1.55)	0.263
Number of Visits in year				
<6 times	Ref		Ref	
≥6 times	1.35 (1.08–1.69)	**0.008**	1.51 (1.13–2.03)	**0.006**
Length of waiting time				
<1 h	Ref		Ref	
1–2 h	0.68 (0.51–0.89)	**0.006**	0.73 (0.52–1.03)	0.075
3–4 h	0.48 (0.34–0.67)	**<0.001**	0.73 (0.47–1.13)	0.153
5–6 h	0.84 (0.61–1.16)	0.284	0.87 (0.58–1.31)	0.504
>6 h	0.68 (0.41–1.11)	0.122	0.71 (0.34–1.45)	0.343
Factor 1 score: Improving values and attitudes	2.32 (2.06–2.6)	**<0.001**	1.28 (1.07–1.53)	**0.008**
Factor 2 score: Cleanliness of clinic	2.47 (2.19–2.79)	**<0.001**	1.45 (1.2–1.75)	**<0.001**
Factor 3 score: Safe and effective care	2.43 (2.16–2.73)	**<0.001**	1.34 (1.13–1.59)	**0.001**
Factor 4 score: Infection control	2.21 (1.98–2.46)	**<0.001**	1.04 (0.85–1.28)	0.679
Factor 5 score: Availability of medicines	5.55 (4.67–6.6)	**<0.001**	4.31 (3.55–5.23)	**<0.001**

Bold faced *p*-values are significant at 5%; italicized *p*-values are significant at 10%.

The multiple logistic regression model was performed by adjusting for all the covariates used in the univariate analysis (Table 4). Adjusting for other variables, patients aged 51 years and above had an increased odds of 3.18 (95% CI:1.31–7.75) of being satisfied compared to those aged 18-30 years. Patients who reported having visited the clinic at least 6 times had 51% increased odds of being satisfied (OR = 1.51, 95% CI:1.13–2.03) adjusting for other variables. The odds of being satisfied increased by 28% (AOR = 1.28, 95% CI:1.07–1.53), 45% (AOR = 1.45, 95% CI:1.2–1.75), 34% (AOR = 1.34, 95% CI: 1.13–1.59) and 4.31 (95% CI: 3.55–5.23) for every score increase in the improving values and attitude factor, cleanliness of clinic factor, safe and effective car factor and availability of medicine factor adjusting for other variables, respectively.

### Model diagnostics for the adjusted model

The post-estimation assessment of the fitted adjusted model showed that the model correctly classified 88.39% of data with an area under the curve of 87.8% which was high. The goodness of fit test showed that the adjusted model had a good fit (*p*-value = 0.6515).

## Discussion

The aims of the study were to investigate the underlying factors influencing patient satisfaction in primary health care amongst chronic disease patients in the City of Johannesburg, South Africa. Generally, high levels of chronic disease patients' satisfaction with care were reported in this study. In addition, the key predictors of patient satisfaction were some sociodemographic factors (age, distance to the clinic, number of visits and waiting times) and key priority areas such as improving values and attitudes, cleanliness of the clinic, waiting time, safety and effective care, availability of medicines has a significant association on overall patient's satisfaction.

Firstly, chronic disease patients' satisfaction exhibits a complex relationship with age, with older patients more likely to have higher satisfaction scores than those younger than 30 years of age. Based on these findings, the lower satisfaction of younger patients may reflect relatively high expectations that cannot be easily met (29). The age-patient satisfaction association was not significant for the 41–50 range. This finding is congruent with studies conducted in high-income settings where older patients report higher satisfaction with services compared to their younger counterparts (30, 31). Middle-aged populations in South Africa are reported to have lower rates of chronic diseases and may not frequently visit healthcare facilities (32), which may explain the non-significant results for this age group.

The results also showed that a distance longer than 5 km to the clinic was markedly associated with patient dissatisfaction; which is similar to previous findings (33). Important to note that in South Africa chronic patients are known to travel up to 60 km or more to access a health facility. Travelling such long distances to clinics is not only time-consuming and tiresome but more importantly, becomes a risk in increasing the severity of the illness as well as death (34). Moreover, there are extra costs in travelling lengthy distances (34, 35), which are not considered within existing frameworks including SERVQUAL as well as the holistic 5P model (15). Donabedian's model explains that when accessing care, patients' negative emotions experienced while seeking care are important contributors to patient dissatisfaction (20).

Surprisingly, it was also shown that frequent users were more likely to be satisfied with chronic disease care than non-frequent users. Precisely, results showed that those who visited the clinic more than >6 times yearly were more satisfied compared to those who visited the primary healthcare facilities less. This finding is in contrast with other studies, as the non-frequent users rated their satisfaction level higher compared to frequent users. In addition, researchers found that patients who already had previous clinical admission tended to be more demanding or critical and had lower satisfaction scores (36). This could be attributed to the fact that patients have adapted to the level of quality of care given. They only know this kind of care, hence high levels of satisfaction. This is supported by Donabadien's model, whereby patients' expectations often predict the level of satisfaction with the service received (20).

Participants placed importance on improving the value and attitudes of chronic disease patients through communication by treating the patient with dignity, informing them about their condition and informing them of the complaints procedure at the particular clinic (37). Patients were particularly satisfied with the physician's advice and treatment, explanation of the patient's condition and their ability to treat the patient with respect and dignity. Good communication between patients and care providers has been described as the single most important component of good medical practice (38). Similarly, previous studies show that respect & politeness, communication skills and technical competence were strong predictors of patient satisfaction and had a positive association (39). The act of communication between nurses, physicians and patients is vital in healthcare, and failure to communicate has been linked with poor quality service provision and patient errors.

Studies have confirmed that physicians can enhance satisfaction by spending minimal time chatting with the patient and by allowing sufficient time for exchange with more understandable explanations and information (36). Thus, physicians can promote higher rates of satisfaction by improving the way of interaction with a physician or PHC nurse who makes effort to address the concerns of patients. This is well aligned with the personnel and physician elements of the 5P model and could inform strategies for improving patient satisfaction. However, it may be important for such a model to be adapted for South Africa to include other health providers such as traditional healers (15). Furthermore, healthcare providers in South Africa are maldistributed across sectors, with many experiencing high workloads, especially within the public sector (40). The consideration of such specific challenges within this context is required when applying a framework like this in improving the quality of care and patient satisfaction.

The factor variable representing the cleanliness of the clinic measured whether there are refuse bins in the waiting area, whether the walls were free of dirt and also whether the floor and tables were clear of splashes, spills and stains which was significantly associated with chronic disease patients' satisfaction with care. This is in line with a study that was conducted in Saudi Arabia, where patient satisfaction stems from cleanliness (41). Patients who perceive clinic cleanliness in a negative light often give low scores on chronic disease patient satisfaction surveys. This, however, contrasts with findings from Nigerian and other low and middle-income countries that investigated patient satisfaction with cleanliness and showed no statistical significance (42). The association between the cleanliness of the clinic and patient satisfaction could be attributed to the possibility that many assume that they are at greater risk of acquiring a healthcare-associated infection during their stay if health facilities are not kept to the best standards of cleanliness. This is supported by research from other low and middle-income countries where satisfaction with the environment emerged as a strong determinant (43). However, there is an indication of this being different for HIV patients and non-HIV patients (44) which may warrant further investigation in future studies. Furthermore, the SERVQUAL framework refers to the neatness and visual appeal of a facility (12) but not specifically cleanliness as operationalized in this study and specific to the South African context.

In the current study, a significant association between waiting time and patient satisfaction was found. Most patients showed a significant level of dissatisfaction with the waiting time. Waiting time provides measures of how long the patient is willing to wait to receive care from the physician, nurse or pharmacy. Chronic disease patients' displeasure with the waiting time in the facility is in line with findings by several studies, which documented the relationship between waiting for service and overall satisfaction, with longer waiting times being associated with decreased patient satisfaction (45). Waiting time remains a challenge in producing quality healthcare services in Johannesburg, South Africa (46). In the context of health service delivery, one major reason that could account for this delay in delivery is various administration processes (paperwork, folder retrieval and filling of health medical aid claim) which takes place before the patient is assisted by the doctors and other health workers. In addition to this, there are large numbers of chronic disease patients attending primary healthcare clinics, however, insufficient doctors to provide the services are employed. The shortage of human resources is a detrimental hurdle affecting the provision of health care services (47). It is, therefore, necessary for frameworks such as the 5P model to be adapted for the South African context, whereby these challenges are accommodated within proposed solutions aimed at improving patient satisfaction.

The study results also show a positive association between patient satisfaction and safety as well as effective care amongst the Johannesburg population. This includes a security guard visible at all times, a visible ramp for wheelchairs, restrooms that are easily accessible to individuals with disabilities and feelings of safety when in the clinic. In terms of safe and effective care, the results of the current study also report a significant proportion who report being completely satisfied with this. The study results are in line with previous findings from high-income settings showing positive association (48). The positive association could be accounted for by the fact that, for the patient to be cured of illness, they need to have easy access to, as well as feel safe at the health facility. When access is poor or health facilities are unsafe, the patient has a higher risk of hospitalization. This contrasts with findings from low and middle-income settings, where lower proportions of patients were satisfied with the ease of accessing care reported as 56% in Benin City and 41.2% in Ibadan (8). However, in the context of South Africa characterised by high crime rates, there is an expectation from patients to be able to access care in a secure environment, which is a critical factor in chronic disease patient satisfaction. The presence of use of a range of security measures like CCTV cameras, alarms, electronic access control systems for doorways and a visible guard could assist patients to feel at ease (49). Existing frameworks, however, do not particularly include an understanding of such context-specific issues and as such provide the generic application of strategies for improving the quality of care and patient satisfaction. Findings such as this are therefore critical for the adaptation of such frameworks in designing appropriate interventions for ensuring safety and security of patients in South Africa, in an effort to improve satisfaction and quality of care provided.

The current study reports that there is a statistical association between patient satisfaction and the availability of medicines. This association could be explained by the fact that patients want to heal rapidly and for that to happen, medications must be administered. Therefore, patients want doctors to communicate with them about medications, especially on use and side effects (50). This has positive effects on their perceptions of pain and responsiveness, which further explains the association with overall patient satisfaction, as shown in previous studies (50). This is in line with Mezemir et al.'s study (51) where patients with the availability of all the prescribed drugs in the hospital pharmacy were 4.30 times more likely to be satisfied. However, it could be argued that conducting satisfaction surveys in health facilities often leads to high satisfaction scores, a bias attributable to fear of victimization or punishment of patients by healthcare providers (1). If the study was conducted far from the health facilities, there is a likelihood of getting completely different results.

The effectiveness of treatment was the most important factor for the selection and recommendation of the clinic, along with adequate information about the disease and explanations about the treatment plans, investigations, effects of medicines and side effects. The national health government of South Africa implemented the ideal clinic realisation and maintenance programme, which was designed in response to the recurring deficiencies in the quality of primary health care services. It has ensured that medicines are always readily available (52). However, it is important to acknowledge that the unavailability of medication has been a major issue faced in many low and middle-income countries that have a high prevalence of chronic diseases like hypertension. In South Africa, the issue of drug stockouts has been mitigated by the implementation of the Central Chronic Medicines Dispensing and Distribution (CCMDD) program to improve access to medicines (53).

Despite this, negative patient experiences characterised by long waiting times and repeat visits are reported due to an overburdened system with low personnel-to-patient ratios. Donabedian's model underpinned by structure, process and outcome posits that the progression through these is sequential and linear. In relation to these findings, this seems appropriate considering that the process of acquiring medicines may be flawed due to is a structural issue such as low staffing levels. This then determines health outcomes for patients who may become dissatisfied with the quality of services received. Process modification through changes in structure could improve outcomes, although this has limitations for contexts where patient characteristics (including sociodemographics, beliefs and preferences), as well as environmental factors (including physical, political and social aspects), are precursors for delivering quality care (20). The study showed limitations with its use of a structured closed-ended questionnaire, which inhibited the respondent's level of expression. Residual confounding factors cannot be ruled out since some of the variables like the number of years patients have been living with the chronic condition were not included in the primary survey. The diverse conceptual understanding of patient satisfaction also means that the study findings are population and setting-specific, although the large sample size of this study allows for the generalisability of findings across similar settings. Nonetheless, a novel method of data analysis, not well utilised in patient satisfaction research indicates the reliability of the measurement tool to capture the determinants of patient satisfaction within the South African context. Although further research using this method is needed, the measurement of patient experiences related to patient satisfaction creates a foundation and an opportunity for building on this research in other similar settings. This has implications for policy and practice towards the improvement of patient experiences such as security and safety, to ensure healthcare quality and service utilization for better chronic disease outcomes in South Africa.

## Conclusions

Key predictors of patient satisfaction were found to be sociodemographic factors including age, distance to the clinic, number of visits and waiting times as well as factors such as improving values and attitudes, cleanliness of the clinic, waiting time, safety and effective care and availability of medicines. When applying existing frameworks to address these for the improvement of patient satisfaction, uptake of health services as well as delivery of quality care in South Africa, it is recommended that some context-specific adjustments be considered for factors such as the safety of patients when receiving care.

## Data Availability

Data is available from the corresponding author upon reasonable request.
